# Editorial: Application of Green Technologies for Recovering Aromatic Compounds From Natural Resources

**DOI:** 10.3389/fnut.2022.945730

**Published:** 2022-06-22

**Authors:** Jelena Vladic, Ana Rita C. Duarte

**Affiliations:** Faculdade de Ciências e Tecnologia, Universidade Nova de Lisboa, Lisbon, Portugal

**Keywords:** aromas, green technologies, clean technologies, alternative solvents, aromatic compounds, extraction, stabilization

The application of aromatic components is highly widespread in different areas. These components account for the sensory characteristics of food, hence they determine, to a significant extent, the acceptability of food by consumers (1). Moreover, aromatic compounds are of high importance in the perfume and cosmetic industries. Apart from sensory characteristics, aromatic components possess medically relevant activities and are therefore of great significance in the pharmaceutical industry (2). Furthermore, it was estimated that the global flavors and fragrances market would increase from $25.89 billion to $36.30 billion between 2021 and 2028 (3). The markets that are driving the demand are food and beverages (35%), fragrances, cosmetics, and aromatherapy (29%), household (16%), and pharmaceutical (15%) (4).

Conventional methods for the isolation of aromatic compounds from natural resources have numerous drawbacks including the use of unsafe solvents, inadequate use of resources, irrational use of energy, low yield, and low quality of the product. Therefore, it is necessary to develop procedures for a more efficient and greener attainment of aromatic components from natural resources.

The scientific papers within this Research Topic present the current state and future perspective of the application of sustainable and clean extractions and solvents to obtain aromatic compounds from natural resources.

Li et al. investigated the procedure of valorization of *Tribute citrus* peels as sources of aromatic components. To obtain essential oil, they applied ultrasound-assisted pre-treatment extraction methods and the traditional method of hydrodistillation. Box-Behnken design was used to optimize the process of obtaining essential oil. Moreover, the impact of the solid-liquid ratio, sodium chloride concentration, and process time on the essential oil yield was investigated. It was determined that ultrasound increased the yield of essential oil by 33.09% with a significant decrease in process time by 40 min. The obtained essential oils possessed D-limonene as the dominant component, followed by α-pinene, sabinene, γ-myrcene, and β-phellandrene.

Ultrasound-assisted extraction was also applied to obtain *Urtica dioica* L. extracts. In the Šic Žlabur et al. study, the adequacy of applying two ultrasound systems—ultrasound probe and ultrasonic bath, was examined. Additionally, the impact of time and ethanol concentration on the extraction efficacy was explored. A significant advantage of the ultrasound-assisted extraction over the conventional extraction was established regarding the higher content of bioactives and higher antioxidative capacity. Furthermore, the application of the ultrasound probe system proved to be considerably more efficient compared to the ultrasound bath, resulting in the intensification of the extraction and the significantly shorter time required to extract the target components.

Arya et al. applied ultrasound- and microwave-assisted extraction to extract seeds of *Celastrus paniculatus*. For comparison, the classical methods of maceration and percolation were also applied. The results showed that optimizing the conditions of the microwave- and ultrasound-assisted extractions achieved a significant reduction in extraction time, improvement in extraction yield, and the concentrations of polyphenols, certain fatty acid esters, alkaloids, sesquiterpenes, and steroidal compounds. Furthermore, the extracts obtained at optimal conditions also exhibited biological activity, improving the behavioral recovery, oxidative stress induced parameters, and acetylcholinesterase concentration.

For the extraction of bioactive components of *Cannabis sativa*, deep eutectic solvents (DES) on the basis of terpenes, sugars, and natural organic acids were used in the Tiago et al. study. Menthol-based solvents were efficient for the extraction of cannabinoids, surpassing the yield of extraction with ethanol. The additional advantage of using DES was also the achievement of higher extraction selectivity. In addition, it was determined that different hydrophobic and hydrophilic DES were adequate for attaining different bioactive components of *C. sativa* such as phenols, flavonoids, and terpenes. Apart from their role as extraction solvents, it was established that DES (Lactic acid:Glucose and Proline:Lactic acid) could also improve the solubility of cannabinoids in aqueous media. Therefore, this study clearly indicated the vast potential of DES, enabling the attainment of natural bioactive components and their more efficient applications.

Hadj Saadoun et al. proposed the fermentation with lactic acid bacteria (LAB) for the valorization of vanilla by-products and attainment of vanillin and other valuable aromatic compounds. The possibility of valorization of two different vanilla by-products was evaluated, with and without seeds, by using eight different LAB strains. Moreover, a comparison between the mono and co-culture of LAB was performed. Fermentation did not affect the concentration of the main volatiles except in the co-cultures where an increase in the concentration of hexanoic acid, hexanol, and guaiacol was recorded. In addition, due to fermentation with LAB, it was determined that there was a change in sensory characteristics. The panelists scored the samples without seeds as less tasty, while the samples with seeds were rated as sweet with vanillin, fatty, and pudding notes. The study suggested that the products obtained by fermentation could be used in the food industry or as materials for further extraction of aromatic components.

The aforementioned studies represent valuable suggestions and green solutions for a more efficient and rational use of natural resources for recovering bioactive and aromatic compounds. Considering the growing demand and limited natural resources, further investigation and development of new procedures and solvents are necessary. Aside from providing scientific data, the collaboration and networking between science and the industry are required to further accelerate the implementation of green processes.

## Author Contributions

All authors listed have made a substantial, direct, and intellectual contribution to the work and approved it for publication.

## Funding

This project has received funding from the European Union's Horizon 2020 Research and Innovation Programme under the Marie Sklodowska-Curie Grant Agreement No. 101003396.

## Conflict of Interest

The authors declare that the research was conducted in the absence of any commercial or financial relationships that could be construed as a potential conflict of interest.

## Publisher's Note

All claims expressed in this article are solely those of the authors and do not necessarily represent those of their affiliated organizations, or those of the publisher, the editors and the reviewers. Any product that may be evaluated in this article, or claim that may be made by its manufacturer, is not guaranteed or endorsed by the publisher.
